# Quantum Corrections Crossover and Ferromagnetism in Magnetic Topological Insulators

**DOI:** 10.1038/srep02391

**Published:** 2013-08-09

**Authors:** Lihong Bao, Weiyi Wang, Nicholas Meyer, Yanwen Liu, Cheng Zhang, Kai Wang, Ping Ai, Faxian Xiu

**Affiliations:** 1 State Key Laboratory of Surface Physics and Department of Physics, Fudan University, Shanghai 200433, China; 2 Department of Electrical and Computer Engineering, Iowa State University, Ames, IA 50010, USA; 3 These authors contributed equally to this work.

## Abstract

Revelation of emerging exotic states of topological insulators (TIs) for future quantum computing applications relies on breaking time-reversal symmetry and opening a surface energy gap. Here, we report on the transport response of Bi_2_Te_3_ TI thin films in the presence of varying Cr dopants. By tracking the magnetoconductance (MC) in a low doping regime we observed a progressive crossover from weak antilocalization (WAL) to weak localization (WL) as the Cr concentration increases. In a high doping regime, however, increasing Cr concentration yields a monotonically enhanced anomalous Hall effect (AHE) accompanied by an increasing carrier density. Our results demonstrate a possibility of manipulating bulk ferromagnetism and quantum transport in magnetic TI, thus providing an alternative way for experimentally realizing exotic quantum states required by spintronic applications.

In recent years, topological insulators (TIs) have received considerable attention due to their novel properties arising from strong spin-orbit coupling and massless Dirac-cone-like surface states that are protected by time-reversal symmetry (TRS)^1^,^2^,^3^,^4^,^5^,^6^,^7^. The exotic surface transport properties are manifested by the prohibition of backscattering events upon non-magnetic perturbations^8^,^9^ and the enhanced quantum corrections of magnetoconductance (MC), namely weak antilocalization (WAL) of Dirac fermions^9^,^10^,^11^. Breaking the TRS in TIs by interfacing a TI material with an insulating ferromagnetic film^12^,^13^,^14^ or simply by magnetically doping^15^,^16^,^17^,^18^ allows for the opening of a surface energy gap and the generation of massive surface carriers^16^. A variety of exotic properties can be realized including the topological magnetoelectric effect^18^,^19^,^20^, the quantized anomalous Hall effect^18^,^21^,^22^,^23^, imaging magnetic monopoles^24^, and the Faraday & Kerr effects in TIs^25^,^26^. Revelation of these topologically non-trivial states calls for a comprehensive understanding of electronic transport response of magnetic doping in TIs.

One signature of such transport response is that when the TI is magnetically doped, a weak localization (WL) effect will naturally emerge as a result of the TRS breaking and the surface state gap opening^27^,^28^,^29^. By tuning the size of the opened surface gap or by moving the position of Fermi energy, the quantum corrections of MC experience a crossover from WAL to a conventional parabolic dependence of magnetic field and finally to WL, which were verified both theoretically^27^ and experimentally^28^. It has been shown that substituting the Bi sites in parent Bi-based tetradymite compounds (Bi_2_Se_3_, Bi_2_Te_3_, Sb_2_Te_3_, Bi_2-x_Sb_x_Te_3_, and Bi_2_Se_3-x_Te_x_) by transition metal ions (Cr, Fe, Mn, V, etc.) will produce a long-range ferromagnetic order either by coupling of local magnetic moments with bulk electron spins through Van Vleck mechanism^21^,^30^ or by Dirac fermion mediation of local magnetic moments via Ruderman-Kittel-Kasuya-Yosida (RKKY) exchange mechanism^15^,^17^,^31^,^32^. A hallmark of the presence of such ferromagnetism is anomalous Hall effect^33^, where Hall resistance shows a hysteresis loop behavior under magnetic field^30^,^31^,^34^. Observations of TRS breaking and surface gap opening by angle-resolved photoemission spectroscopy (ARPES)^16^,^35^,^36^,^37^,^38^,^39^,^40^ accelerate the investigations of transport properties in the magnetically doped TIs. Recently the Dirac fermion mediated ferromagnetism via RKKY interaction was observed in Mn-doped Bi_2_Te_3-y_Se_y _nanpoflakes^31^ and carrier-independent long range ferromagnetic order was confirmed in Cr-doped Bi_2-x_Sb_x_Te_3_ thin films^30^, in which a large Van Vleck-type of spin susceptibility was established between local magnetic moments and bulk electrons that guarantees the robust ferromagnetism. However, to date, the tuning of ferromagnetism upon different dopant concentrations has not yet been demonstrated and the doping effect on the ferromagnetism needs to be further explored in the entire composition range. Recent experimental observation of quantum anomalous Hall effect in a magnetic TI Cr-doped Bi_2-x_Sb_x_Te_3_ thin films also highlighted the role of different amount of dopants played in tuning the transport properties^23^.

In the present study, we report on the manipulation of ferromagnetism and quantum corrections in Cr_x_Bi_2-x_Te_3_ thin films grown on mica by varying the Cr doping concentration. In the low doping regime (x ≤ 0.14), the quantum corrections of MC experience a crossover from WAL to WL. Once the ferromagnetism emerges, the quantum corrections of MC are dominated by the WL effect. While in the high doping regime (x ≥ 0.14), a monotonic enhancement in anomalous Hall effect was observed upon increasing Cr doping concentration. Our results demonstrated a promising way to manipulate the transport behavior of magnetic topological insulators.

## Results

### Structural characterizations of MBE grown Cr_x_Bi_2-x_Te_3_ thin films

**Figure 1:**
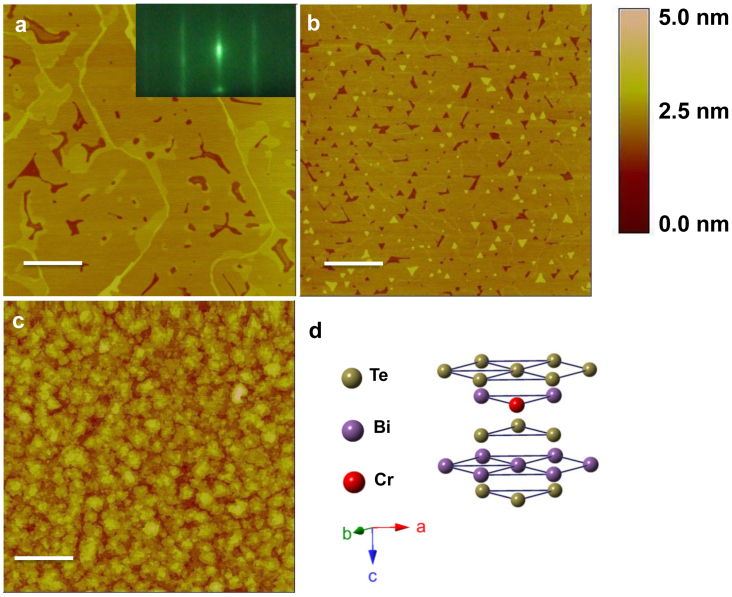
Structural characterizations of MBE grown Cr_x_Bi_2-x_Te_3_ thin films on mica. (a) An AFM image of a pure Bi_2_Te_3 _(x = 0) thin film, indicating an atomically smooth surface and micrometer-sized terraces. Inset is a representative RHEED pattern from the smooth surface of Bi_2_Te_3_ thin films. The streaky lines revealed the single crystalline nature of the films and a layer-by-layer growth mode. (b) An AFM image of Cr_0.08_Bi_1.92_Te_3_ thin film, showing lightly doping of Cr induced the nucleation of triangular-shaped terraces on the smooth surface without roughening the surface. (c) An AFM image of Cr_0.27_Bi_1.93_Te_3_ thin film, demonstrating heavily doping of Cr roughed the smooth surface with an average surface roughness of ~1 nm. (d) Atomically structural model of Cr_x_Bi_2-x_Te_3_, showing the position of Cr impurities is located at Bi sites of the quintuple layer. Scale bars are 1 μm.

Using molecular beam epitaxy (MBE) with high purity source materials, 15 quintuple layers (QLs) Cr_x_Bi_2-x_Te_3_ thin films were grown on muscovite mica via van der Waals epitaxy under a large range of dopant concentrations, where the Cr cell temperature was varied from 1020 to 1220°C. During the growth the surface quality was *in-situ* monitored using reflection high-energy electron diffraction (RHEED) technique. Figs. 1a–c show the representative AFM images of as-grown pure (Fig. 1a) and Cr-doped (Figs. 1b and 1c) Bi_2_Te_3_ thin films. The pure Bi_2_Te_3_ films have an atomically flat surface with micrometer-sized terraces, indicating the high crystalline quality (Fig. 1a). RHEED was used to monitor the *in-situ* growth dynamics with the electron beam incident to the 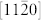 direction. The sharp streaky lines in the inset of Fig. 1a indicate a layer-by-layer 2D growth mode and a flat surface morphology. Lightly doping with Cr (x = 0.08) resulted in the formation of triangular-shaped terraces without roughening the flat surface (Fig. 1b). While heavily doping with Cr (x = 0.27) roughened the surface with a root-mean square (RMS) roughness of ~1 nm. Previous studies on the Cr-doped TI materials Sb_2_Te_3_ and Bi_2_Se_3_ with rhombohedral symmetry have shown a tendency for Cr to be incorporated into the Sb or Bi sublattice^41^,^42^. The Cr in Bi_2_Te_3_ would likely be incorporated into Bi sites of the quintuple layer, which is schematically shown in Fig. 1d. The roughened surface of heavily Cr-doped Bi_2_Te_3_ thin films is probably due to the competition between Cr atoms and Bi atoms at Bi sites in quintuple layered structure during the growth^34^. The high quality MBE grown thin films facilitate the revelation of transport response of Cr doping in Bi_2_Te_3_.

### Crossover of quantum corrections in lightly doped Cr_x_Bi_2-x_Te_3_ thin films (x ≤ 0.14)

**Figure 2:**
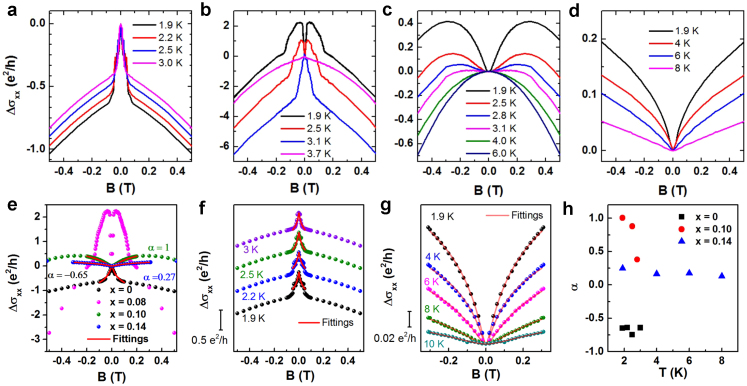
Crossover of quantum corrections of magnetoconductance (MC) with increasing Cr content in Cr_x_Bi_2-x_Te_3_ thin films (x ≤ 0.14). (a) MC curves of pure Bi_2_Te_3_ thin films, showing the negative MC features of WAL. (b) MC curves of Cr_0.08_Bi_1.92_Te_3_ thin film, indicating a non-monotonic behavior with sharp downward cusp at low temperatures (< 3.1 K) and re-presence of WAL at higher temperatures (3.1 and 3.7 K). (c) MC curves of Cr_0.10_Bi_1.90_Te_3_ thin film, showing a crossover from downward cusp feature to parabolic dependence with increasing temperatures. (d) MC curves of Cr _0.14_Bi_1.86_Te_3_ thin film shows a WL dominated behavior. (e) HLN model fitting of MC curves with different Cr content at temperature of 1.9 K. (f) and (g) HLN model fitting of MC curves of pure Bi_2_Te_3_ and heavily doped Cr_0.14_Bi_1.86_Te_3_ thin films, showing that both WAL and WL can be fitted well to the HLN model. (h) Pre-factor of α in HLN model of thin films with different Cr concentrations.

The Cr-doped Bi_2_Te_3_ films on mica were etched into the Hall bar geometry using reactive ion etching (RIE) and the low temperature transport measurements in the longitudinal and transverse directions were carried out with a physical properties measurement system (PPMS) when a current is applied along the Hall bar and a magnetic field is applied perpendicularly to the surface. The longitudinal MC at low temperatures for a series of Cr doping concentrations is shown in Figure 2. We see evidences of the incorporation of Cr into the lattice and its effect on the transport for low Cr doping concentrations (x ≤ 0.14) via quantum corrections. In pure Bi_2_Te_3 _thin films a sharp upward cusp is shown in the MC curve at low magnetic fields (Fig. 2a), indicating a WAL behavior^9^,^10^,^11^,^43^. This has been identified as a key feature of topological surface states, where Dirac fermions travel around a self-intersecting path or loop due to the spins rotating in opposite ways for the different path directions and a π Berry's phase is accumulated^10^,^11^,^44^. The destructive interference due to π Berry's phase leads to an enhancement of MC. Applying an external magnetic field suppresses the destructive interference, giving rise to a negative MC^11^,^44^,^45^. One interesting question to ask is what if the magnetic impurities are incorporated into the TI materials? Theoretical predictions suggested that when doping with magnetic impurities, a competing WL effect will be introduced and the localization behavior is a result of the competition between WAL and WL^27^,^29^,^46^,^47^. For the slightly doped sample (x = 0.08, Fig. 2b), at T = 1.9 K the sharp upward cusp feature of WAL is gone and the magnetoconductance exhibits a non-monotonic increase with the increase of magnetic field, where a sharp downward cusp is developed at small magnetic fields. When the temperature warms up to 3.1 K, the non-monotonic behavior disappears and the WAL shows up again. Further increasing the temperature to 3.7 K flattens the cusp feature, indicating that the WAL is weakened and it can only survive in a small temperature range. For the sample doped with x = 0.10 (Fig. 2c), WAL is completely suppressed and a non-monotonic behavior is presented up to 3.1 K before a classical parabolic dependence of the magnetic field (~B^2^) of the MC appears around 4 K (Supplementary materials Fig. S1). Further increasing the doping concentration to x = 0.14 (Fig. 2d), downward cusp feature is persistent up to 10 K, indicative of a WL dominated behavior.

The quantum corrections to the 2D MC can be described by the Hikami-Larkin-Nagaoka (HLN) model^48^ and is given analytically by the equation 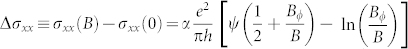, where 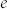 is the electron charge, 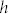 is Planck's constant, 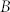 is the magnetic field, 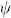 is the digamma function, and 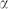 is a coefficient whose value is determined by the nature of the corrections being WL or WAL, or having contributions from both effects. Additionally, we have 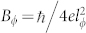 in which the coherence length is characterized by 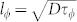, 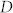 is the diffusion coefficient and 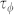 is the dephasing time. The undoped samples show a WAL behavior (Fig. 2a) and can be fitted well to the HLN model (Figs. 2e and 2f). The resultant α value ranges from −0.65 to −0.75 (black squares in Fig. 2h) with increasing temperatures, consistent with the typical values of WAL originated from 2D surface states of TI^11^,^49^,^50^,^51^. And for the heavily doped samples with x = 0.14, the MC has an excellent fit to the HLN model (Fig. 2g) with α values from 0.25 to 0.09 (blue triangles in Fig. 2h) suggesting a typical WL behavior^28^,^51^,^52^,^53^. However, the fit becomes challenging for the lightly (x = 0.08, Fig. 2b) and intermediate doping (x = 0.10, Fig. 2c) samples, primarily because of the competition between WAL and WL. Under these circumstances, the weight ratios of competing terms of WAL and WL are difficult to be extracted. Nevertheless, in low magnetic fields (−0.3 T < B < 0.3 T), the sample with intermediate doping yields α values ranging from 1.0 to 0.37 with increasing temperatures (T ≤ 2.8 K) as opposed to a large deviation from the HLN model at high fields (for B > 0.3 T, supplementary Fig. S1)^27^.

It has been proposed that the opening of the surface energy gap from the TRS breaking is responsible for this crossover from WAL to WL^27^,^28^. Experimental observation in Cr_x_Bi_2-x_Te_3_ thin films showed that with x = 0.23, the surface states were completely suppressed. Correspondingly the system became a dilute magnetic semiconductor (DMS)^28^. It is well known that the incorporation of magnetic impurities leads to the increased disorder in the films causing localization in the electronic states, known as WL, which is strongly related to field-induced magnetization^29^. In our scenario, with a much lower Cr doping of x = 0.14, the MC is completely governed by the WL effect as opposed to the crossover behavior from WL to unitary parabola with x = 0.10. This suggests that a long-range ferromagnetic order is developed upon the alignment of magnetic moments at low temperatures and low magnetic fields^34^.

### Ferromagnetism in heavily doped Cr_x_Bi_2-x_Te_3_ thin films (x ≥ 0.14)

**Figure 3:**
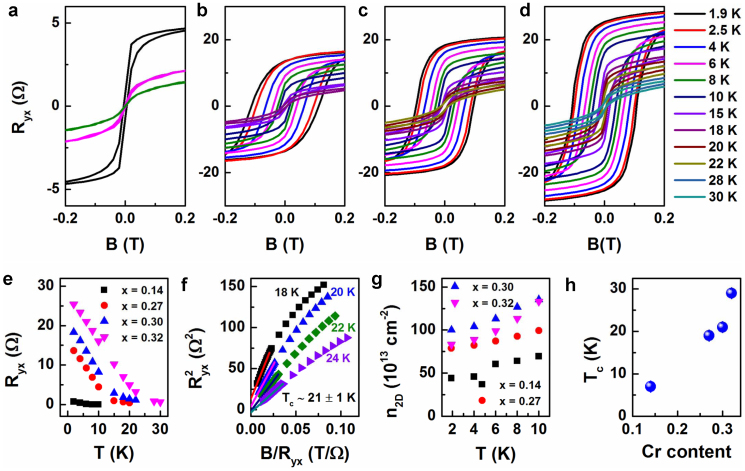
Anomalous Hall effect (AHE) in heavily doped Cr_x_Bi_2-x_Te_3_ thin films (x ≥ 0.14). Quasi-rectangular shaped hysteresis loop in magnetic field dependent Hall resistance curves at low magnetic fields (~0.2 T) in (a) x = 0.14, (b) x = 0.27, (c) x = 0.30, and (d) x = 0.32, showing that a ferromagnetic order is developed in these thin films. Increasing Cr concentration results in an enhancement of the AHE effect. (e) Temperature-dependent Hall resistances R_yx_ of Cr_x_Bi_2-x_Te_3_ thin films at zero magnetic fields. (f) The Arrott plot of the Cr_0.30_Bi_1.70_Te_3_ thin film, showing the polarity change of intercept with increasing temperatures, by which the Curie temperature can be extracted. (g) Temperature-dependent carrier concentration of Cr_x_Bi_2-x_Te_3_ thin films. (h) Curie temperatures of Cr_x_Bi_2-x_Te_3_ thin films with different Cr concentration.

The Hall measurements were carried out to investigate the ferromagnetism in the Cr_x_Bi_2-x_Te_3_ films. In general, the Hall resistance of our samples doesn't show anomaly or hysteresis behavior until the doping level x reaches 0.14. As shown in Figure 3, the Hall resistance R_yx_ displays hysteresis loops resulting from anomalous Hall effect (AHE) at low temperatures^33^, showing a signature of a long-range ferromagnetism^30^,^31^,^34^. The Hall resistivity in a magnetic sample is given by 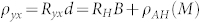, where the first term is the ordinary Hall resistivity and the second term is the anomalous Hall contribution that arises from the magnetization of the material. Here, 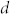 is the thickness of the film, 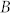 is the applied magnetic field (in Tesla), 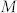 is the magnetization, and 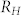 is the ordinary Hall coefficient. Figs. 3 a–d show quasi-rectangular shaped hysteresis loops of Cr_x_Bi_2-x_Te_3_ thin films at low temperatures with x = 0.14, 0.27, 0.30, 0.32, respectively. Both the saturation Hall resistance and the magnetization switching field decrease with increasing temperature, which is commonly observed in ferromagnetic materials. Fig. 3e shows the temperature-dependent R_yx_ of Cr_x_Bi_2-x_Te_3_ films at zero magnetic field. The Curie temperature can be defined as the temperature at which R_yx_ reduces to zero when B = 0 T^30^,^31^. However, R_yx_ does not completely vanish (several of tenth Ohms) at the measured temperature range (Figs. 3 a–e). Therefore, the Curie temperature cannot be simply inferred from the R_yx_-T relationship. The remaining hysteresis behavior can be ascribed to the defect or impurity states from mica substrates, which was previously observed in Mn_x_Bi_2-x_Te_3_ thin films grown on GaAs substrate^34^. Alternative approach to identify the Curie temperature 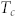 is to use Arrott plots, where 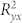 is plotted against 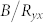 and the extrapolated intercept is proportional to the saturation magnetization (Supplementary material Figure S4)^31^,^34^. The Curie temperature 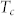 can be extracted when the intercept on the 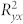 axis goes to zero^31^, as is shown in Fig. 3f. Fig. 3h summarizes the Curie temperature 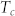 as a function of the Cr doping concentration. The monotonic increase in 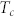 suggests an enhanced AHE with Cr concentration. By examining the Hall resistivity at large magnetic fields where magnetization is saturated and the response of the resistivity to the magnetic field is linear, we can extract the carrier concentration of the samples using Hall coefficient 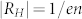, where 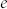 is the electron charge and 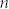 is the carrier concentration. The sign of the ordinary Hall coefficient exclusively shows an *n*-type conductivity for all Cr doping concentrations. Fig. 3g shows a general trend of the increase of the sheet carrier concentration as a function of Cr concentration. The high bulk carrier concentration of 10^14 ^~ 10^15^ cm^−2^ with x = 0.30 ~ 0.32 of Cr indicates that Cr doping generated free carriers in Bi_2_Te_3_. As previously reported, Cr-doped *p*-type Sb_2_Te_3_ crystal exhibited a reduced hole density compared with a pure Sb_2_Te_3_ crystal, suggesting an *n*-type doping nature of Cr^54^. The high doping concentration of Cr will inevitably induced the chemical potential disorder^17^ in the Cr_x_Bi_2-x_Te_3_ thin films due to the competition between Cr atoms and Bi atoms in occupying the Bi sites^34^. The high carrier concentration in Cr_x_Bi_2-x_Te_3_ thin films most likely results from this chemical potential disorder^17^.

**Figure 4:**
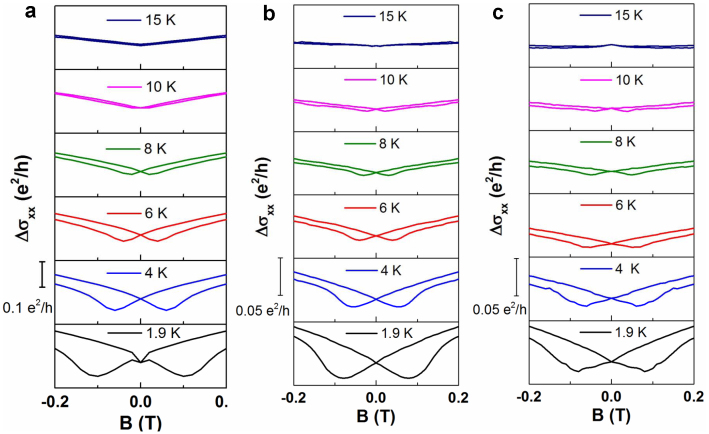
Magnetoconductance (MC) curves of heavily doped Cr_x_Bi_2-x_Te_3_ thin films (x ≥ 0.14). The hysteresis loop behavior in MC curves in (a) x = 0.27, (b) x = 0.30, and (c) x = 0.32, further confirming the establishment of ferromagnetism in these thin films. The MC minima in the MC curves reflect the strength of coercive force. Once x reaches 0.27, the coercive force shows almost no response to the increasing Cr concentration.

As discussed above, once the ferromagnetic order is established throughout the film, the MC is dominated by the WL (x = 0.14). With increasing Cr concentration (Figure 4, (a) x = 0.27, (b) x = 0.30, and (c) x = 0.32), the MC curves show a hysteresis behavior at low temperatures and progressively evolved into trivial flat shape with increasing temperatures. The butterfly-shaped hysteresis loop is an indication of the ferromagnetism in these thin films^31^,^34^,^55^. The MC curves show a negative quantum correction at low magnetic fields^31^,^34^ and the two MC minima presented in each butterfly pattern correspond to the coercive force. Consistent with the AHE results, coercive force varies a small amount with Cr concentrations increasing from x = 0.27 to 0.32 at 1.9 K. Both longitudinal and transverse transport measurements verify the establishment of a long range ferromagnetic order in the heavily doped Cr_x_Bi_2-x_Te_3_ thin films. However, the high carrier concentration observed in these thin films is an obstacle in obtaining the QAH state.

## Discussion

In summary, mica serves as a suitable substrate to create high-quality flat surfaces for the TI material Bi_2_Te_3_. The incorporation of Cr dopants into the Bi_2_Te_3_ produces sufficient disorder to prompt a transition from WAL to WL in MC. The Hall resistivity shows hysteresis loops for Cr doping level x ≥ 0.14 due to the anomalous Hall effect, indicating that the film can become magnetized with a large Cr concentration. The results consolidate the idea that Cr-doping is an appropriate approach to break TRS in the Bi_2_Te_3_ system as predicted in theoretical proposals^21^. The increased carrier concentration with increasing Cr concentration suggests that introducing Cr in Bi_2_Te_3_ thin films indeed generates free carriers. The high carrier concentration (10^14^–10^15^ cm^−2^) in the ferromagnetic Cr_x_Bi_2-x_Te_3_ thin films eliminates the surface state transport and correspondingly rules out the possibility of Dirac fermion mediated RKKY mechanism. Previous experimental findings suggest that the Van Vleck mechanism is characterized by a carrier-independent long range ferromagnetic order^30^. Our experimental results didn't show a direct relation between the carrier density and ferromagnetism. Experimentally the other feature of Van Vleck mechanism is the linear relationship between Curie temperature and magnetic dopants concentration^30^. However, our experiments didn't show a rigid linear relationship between Curie temperature and Cr doping concentration, as is shown in Fig. 3h. Our results demonstrate a clear crossover of quantum corrections of MC from WAL to WL at lightly doped regime (x ≤ 0.14) and conventional butterfly patterned hysteresis loops at heavily doped regime (x ≥ 0.14). The transport signatures from the TRS-broken magnetic topological insulators in this study provides a critical reference for accessing of the gapped surface states, which is an important step toward the realization of novel topological magnetoelectric devices using non-trivial electronic states.

## Methods

### Thin film growth

High-quality crystalline thin films of Cr-doped Bi_2_Te_3_ were grown on freshly cleaved muscovite mica via molecular beam epitaxy in an ultrahigh vacuum system with a base pressure of ~10^−10^ Torr by co-evaporating high purity chromium (99.999%), bismuth (99.999%), and tellurium (99.999%) sources under a Te rich condition. The films were obtained with a Bi cell temperature of 520°C, a Te cell temperature of 320°C, and a substrate temperature between 245 and 275°C. Films were grown with various Cr concentrations by varying the Cr cell temperature from 1020 to 1230°C. The thicknesses of thin films are determined by growth time and flux of Bi, Te, and Cr sources. Typical growth rate is ~0.5 QL/min.

### Characterization

The morphology of as-grown Cr-doped Bi_2_Te_3_ thin films was characterized with an atomic force microscope (AFM, Digital Instruments Nanoscope IIIa) and the Cr-doping profile was analyzed by an Oxford instrument Aztec X-ray energy-dispersive spectrum (EDS) system equipped on a field-emission gun SEM (FEI Quanta 250). At the Cr cell temperatures lower than 1160°C, the yielding Cr doping profile is beyond the detection limit of EDS and the Cr concentrations were inferred from calibrated flux ratios of Cr cell and Bi cell combined with EDS^56^.

### Device fabrication and transport measurement

Standard Hall bar devices of thin films were fabricated by photolithography combined with reactive ion etching (RIE). Ohmic contacts were established using room temperature cured silver paste. The transverse and longitudinal resistances were then measured using a Quantum Design physical properties measurement system (PPMS) that can sweep magnetic fields from −9 T to +9 T at temperatures as low as 1.9 K.

## Author Contributions

F.X. conceived the idea and supervised the overall research. L.B. and N.M. designed and performed the experiments. N.M. and K.W. did the MBE growth of thin films. L.B. fabricated Hall bar devices. F.X., W.W., Y.L., C.Z. and P.A. carried out low-temperature transport measurements. L.B. and N.M. did the data analysis. L.B., N.M. and F.X. wrote the paper with helps from all other co-authors. L.B., W.W. and N.M. contributed equally to this work.

## Supplementary Material

Supplementary Information Supplementary
