# Supplementary material for: Quantum Corrections Crossover and Ferromagnetism in Magnetic Topological Insulators
Source: Sci Rep. 2013 Aug 9;3:2391. doi: 10.1038/srep02391 (PMC3739003; doi:10.1038/srep02391)
Supplement: Supplementary Information — Supplementary [file srep02391-s1.pdf]

**Supplementary materials**

# Quantum Corrections Crossover and Ferromagnetism in Magnetic Topological Insulators

Lihong Bao[1,2*], Weiyi Wang[1,*], Nicholas Meyer[2,*], Yanwen Liu[1], Cheng Zhang[1], Kai Wang[1,2], Ping Ai[1], Faxian Xiu[1,†]

[1]*State Key Laboratory of Surface Physics and Department of Physics, Fudan University, Shanghai 200433, China*

[2]*Department of Electrical and Computer Engineering, Iowa State University, Ames, IA 50010, USA.*

[*]These authors contributed equally to this work.

[†]Corresponding author, Professor Faxian Xiu E-mail: Faxian@fudan.edu.cn Tel: 011-86-21-51630277

**Content**

**S1. Fitting of the magnetoconductivity curves to the HLN model.**

**S2. Temperature-dependent resistance of $Cr_xBi_{2-x}Te_3$ thin films.**

**S3. Magnetic property and Hall effect of $Cr_{0.10}Bi_{1.90}Te_3$ thin film.**

**S4. Arrott plots of ferromagnetic $Cr_xBi_{2-x}Te_3$ thin films.**

**S1. Fitting of the magnetoconductivity curves to the HLN model**

As discussed in the main text, the quantum correction to the 2D magnetoconductance can be described by the Hikami-Larkin-Nagaoka (HLN) model[1]. For pure $Bi_2Te_3$ thin films, the featured negative quantum corrections can be well fitted to the HLN model and this yields a typical α value of -0.65~-0.75 (See the main text, Fig. 2h). For the heavily doped $Cr_{0.14}Bi_{1.86}Te_3$ thin films, however, α is changed to 0.25~0.09, reflecting a typical WL behavior in a ferromagnetic state. . For the slightly doped samples (x = 0.08), at temperatures below 3.1 K, the MC curves show a nonmonotonic behavior, which can't be analytically fitted to the HLN model due to the competing WL/WAL contributions. While at a temperature of 3.1 K and higher, the sharp upward cusp feature appears again and the MC curves can be described as the WAL behavior at small magnetic fields, as is shown in Fig. 1S(a). Due to the competing WL contribution, WAL can only survive in a small temperature range and at small magnetic fields, evidenced by flattened cusp at 3.7 K (Fig. 1S(a)). For intermediate doped samples (x = 0.10), the WAL effect is completely suppressed and the MC curves show a crossover from WL effect to a classical parabolic ($\sim B^2$) dependence with increasing temperatures, as shown in Fig. 1 S(b) and 1 S(c), respectively. From Fig. 1 S (b) and Figure 2c in main text, we can see that the WL effect can only exist at 2.8 K or lower tempearuter. At higher temperatures, the WL effect will evolve into a flat unitary regime and then to classical parabolic behavior. Our results unambiguously demonstrate the evolution of quantum corrections of MC with increasing Cr doping concentrations which coincides with theoretical predictions[2] and other experimental observation[3,4].

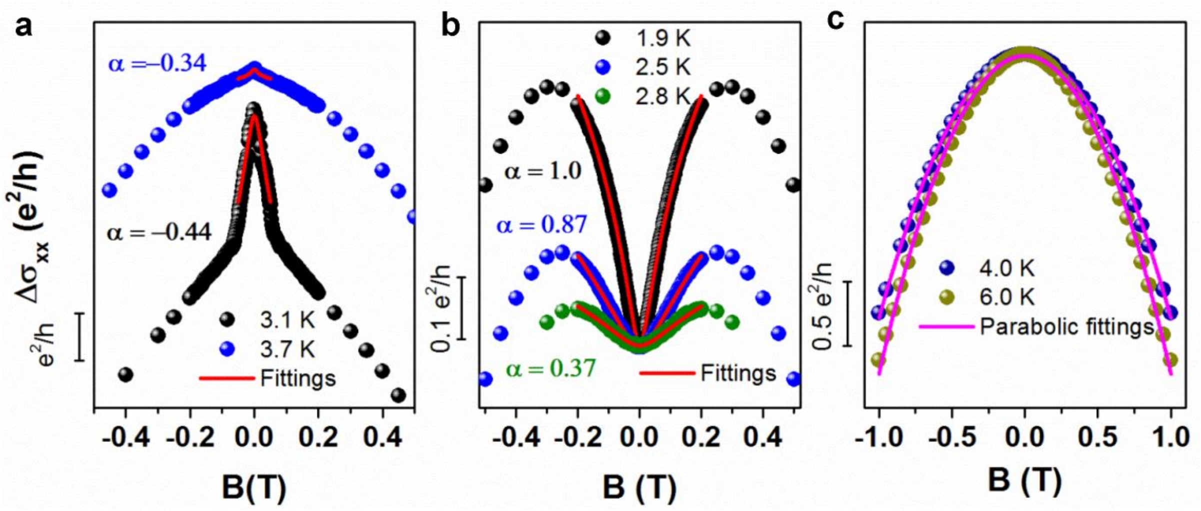


**Figure S1| Fitting of MC curves of $Cr_xBi_{2-x}Te_3$ thin films to the Hikami-Larkin-Nagaoka (HLN) model**. (a) For x = 0.08, at temperatures below 3.1 K (the main text 1.9 K, 2.5 K), MC curves can't be fitted to the HLN model due to the nonmonotonic behavior and WAL feature appears again at 3.1 K, fitting to the HLN model yields a typical value of WAL. (b) For x= 0.10, at T< 2.8 K, the low field MC curves can be fitted, showing a WL feature. (c) At T> 4 K, the MC curves show a classical behavior with parabolic ~$B^2$ dependence.

**S2. Temperature-dependent resistance of $Cr_xBi_{2-x}Te_3$ thin films**

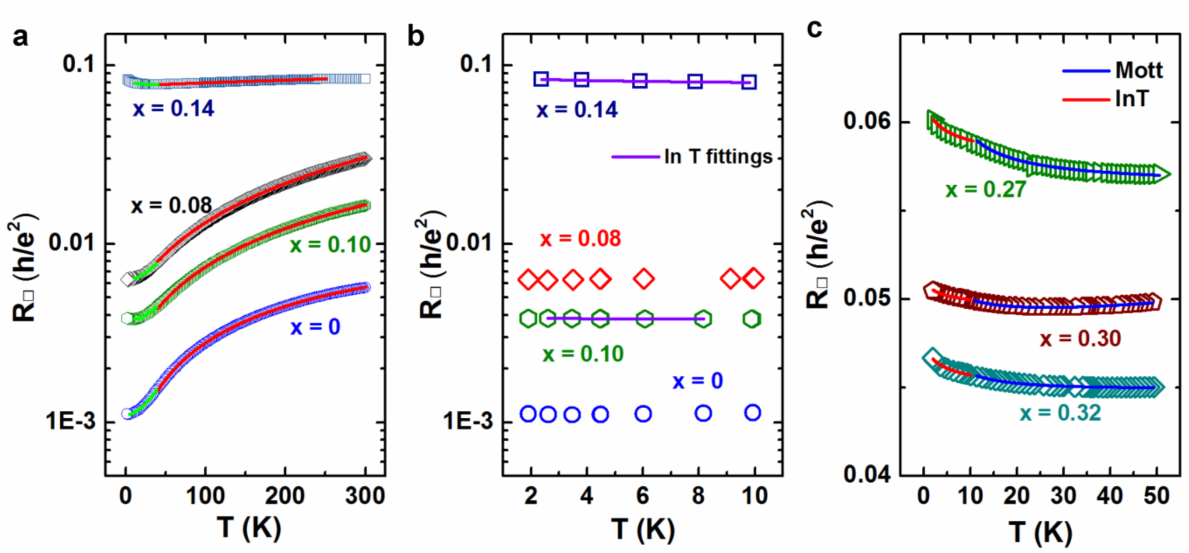


**Figure S2| Temperature-dependent sheet resistance in $Cr_xBi_{2-x}Te_3$ thin films.** (a) *R-T* curves of lightly doped samples (x ≤ 0.14), showing non-monotonic increment in resistance with increasing Cr concentrations. All curves show a metallic behavior at temperatures over 40 K. (b) *R-T* curves of lightly doped samples at low temperatures (< 10 K), indicating that only higher Cr concentration can get a ground insulating state (lnT dependence). (c) *R-T* curves of heavily doped samples (x ≥ 0.27) at low temperatures (< 50 K), showing a monotonic decrement with increasing Cr concentration.

Figure S2 shows the temperature-dependent resistances of $Cr_xBi_{2-x}Te_3$ thin films. As is shown in Fig. S 2(a), in low doping regime (x ≤ 0.14), the sheet resistances show a nonmonotonic increase with increasing Cr concentration, while in high doping regime (x ≥ 0.27), the sheet resistances show a monotonic decrement with increasing Cr concentration. Detailed analysis of the R-T curves suggests that at higher temperatures (T ≥ 40 K) the sample exhibits a metallic behavior which can be fitted with the formula $R = R_0 + a_1T + a_2T^{3/2}$ (red curves in Fig. S 2(a)), where the linear term $a_1T$ corresponds to metallic electron-phonon scattering effect[5,6] and $a_2T^{3/2}$ comes from the ionized impurities effect[6,7]. For pure and lightly doped samples (x = 0, 0.08, 0.10), at low temperatures (10 K ≤ T ≤ 40 K) the R-T curves fit well to

the formula $R = R_0^{'} + b_1T^2$ (green curves in Fig. S 2(a)), where the term $b_1T^2$ corresponds to the electron-electron scattering interactions[8]. As discussed in the main text, when the doping concentration reaches 0.14, the magnetoconductacne curves are governed by WL behavior and ferromagnetism is established in the thin film. Correspondingly, the *R-T* curve of this thin film can be described as a Mott insulating behavior[6], $R : \exp(T_0/T)^{1/4}$. Further increasing the Cr concentration yields a upturn in the R-T curves. Before the onset of upturn, the *R-T* curves are dominated by the Mott insulating behavior (Red curves in Fig. S 2(c)), ascribed to a variable range hopping (VRH) transport behavior in the ferromagnetic $Cr_xBi_{2-x}Te_3$ thin films. At ultralow temperatures (< 10 K), for the thin films with Cr concentration over 10%, the R-T curves show a logarithmic dependence (Fig. S 2(b) and 2(c)), $R : \ln T$, suggesting that a ground insulating state may exist in these thin films[6,9], which shows the tendency of approaching ferromagnetic insulating ground state[10].

## S3. Magnetic property and Hall effect of $Cr_xBi_{2-x}Te_3$ thin film

Both field-dependent magnetization measurement and Hall measurement were employed to confirm that at $x \leq 0.10$, the ferromagnetic order is not developed in the Cr-doped $Bi_2Te_3$ thin films. Fig. S 3(a) shows paramagnetic signals from the sample. Hall resistance also shows a linear dependence with the magnetic field, further confirmed the para-state when $x \leq 0.10$ (Fig. S 3(b)). Fig. S 3(c) shows the M-H curves of $Cr_xBi_{2-x}Te_3$ thin films ($x \geq 0.14$). The hysteresis loops in these curves confirms the presence of ferromagnetic order in these films, which is consistent with AHE effect in these thin films as is shown in the main text (Figure 3).

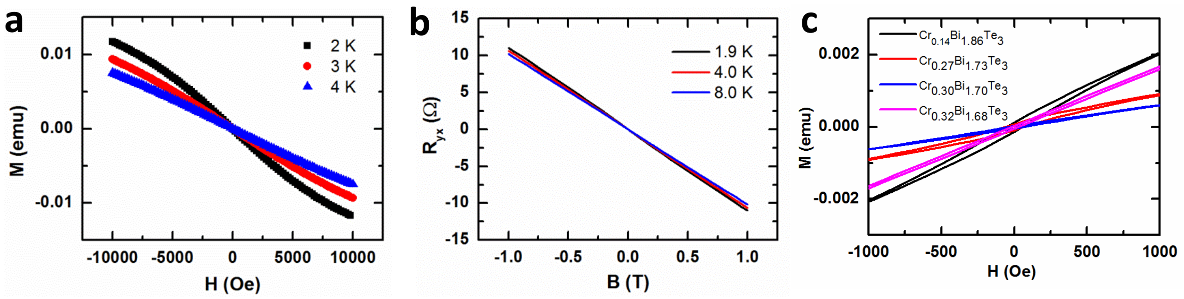


**Figure S3| Magnetic property and Hall effect of $Cr_{0.10}Bi_{1.90}Te_3$ thin film**. (a) M-H curves of $Cr_{0.10}Bi_{1.90}Te_3$ thin film, showing a paramagnetic state. (b) Linear relationship between Hall resistance and magnetic field confirms that no ferromagnetic phase is formed in the sample. (c) M-H curves of $Cr_xBi_{2-x}Te_3$ thin films ($x \geq 0.14$). The hysteresis loops confirms the presence of ferromagnetic order in these films.

## S4. Arrott plots of ferromagnetic $Cr_xBi_{2-x}Te_3$ thin films

As discussed in the maintext, the Hall resistance $R_{yx}$ reflects the magnetization of ferromagnetic materials in AHE. The Curie temperature $T_c$ can be extracted using Arrott plots ,where $R_{yx}^2$ is plotted against $B/R_{yx}$ and the extrapolated intercept is proportional to the saturation magnetization[4,11]. The Curie temperature $T_c$ is defined as the temperature at which the plot yields a straight line with zero intercept. Figure S4 shows the Arrott plots of ferromagnetic $Cr_xBi_{2-x}Te_3$ thin films. The Curie temperature is then determined by the critical temperatures at which the sign of intercept changes polarity.

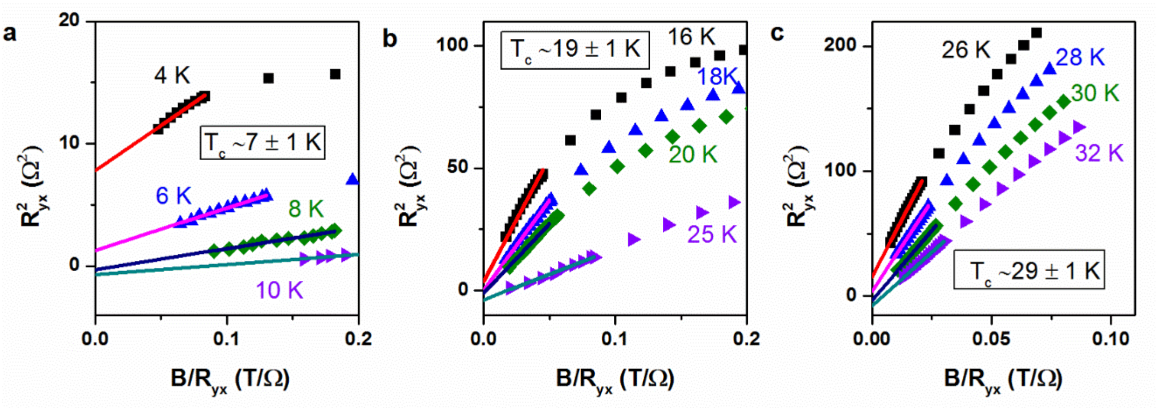


**Figure S4| Arrott plots of ferromagnetic $Cr_xBi_{2-x}Te_3$ thin films.** (a) x = 0.14. (b) x = 0.27. and (c) x = 0.32. The curves at which temperatures show a transition from positive intercept to negative intercept determines the Curie temperature $T_c$.

## References


1. Hikami, S., Larkin, A.I., & Nagaoka, Y., Spin-orbit interaction and magnetoresistance in the two dimensional random system *Prog. Theor. Phys.* 63 (2), 707-710 (1980).
2. Lu, H.-Z., Shi, J., & Shen, S.-Q., Competition between Weak Localization and Antilocalization in Topological Surface States. *Phys. Rev. Lett.* 107 (7), 076801 (2011).
3. Liu, M. *et al.*, Crossover between Weak Antilocalization and Weak Localization in a Magnetically Doped Topological Insulator. *Phys. Rev. Lett.* 108 (3), 036805 (2012).
4. Zhang, D. *et al.*, Interplay between ferromagnetism, surface states, and quantum corrections in a magnetically doped topological insulator. *Phys. Rev. B* 86 (20), 205127 (2012).
5. Checkelsky, J.G., Hor, Y.S., Cava, R.J., & Ong, N.P., Bulk Band Gap and Surface State Conduction Observed in Voltage-Tuned Crystals of the Topological Insulator $Bi_2Se_3$. *Phys. Rev. Lett.* 106 (19), 196801 (2011).
6. Lv, L. *et al.*, Enhanced contribution of surface state and modification of magnetoresistance in $Fe_xBi_{2-x}Se_3$ topological insulator crystals. *J. Appl. Phys.* 113, 043923 (2012).
7. Carle, M., Pierrat, P., Lahallegravier, C., Scherrer, S., & Scherrer, H., Transport properties of n-type $Bi_2(Te_{1-x}Se_x)_3$ single crystal solutions ($x \leqslant 0.05$). *J. Phys. Chem. Solids* 56 (2), 201-209 (1995).
8. Appel, J., Effect of electron - electron scattering on the electrical and thermal conductivity of metals. *Philos. Mag.* 8 (90), 1071-1075 (1963).
9. Liu, M. *et al.*, Electron interaction-driven insulating ground state in $Bi_2Se_3$ topological insulators in the two-dimensional limit. *Phys. Rev. B* 83 (16), 165440 (2010).
10. Yu, R. *et al.*, Quantized Anomalous Hall Effect in Magnetic Topological Insulators. *Science* 329 (5987), 61-64 (2010).
11. Checkelsky, J.G., Ye, J., Onose, Y., Iwasa, Y., & Tokura, Y., Dirac-fermion-mediated ferromagnetism in a topological insulator. *Nat. Phys.* 8 (10), 729-733 (2012).
